# Supplementary material for: Motor learning- and consolidation-related resting state fast and slow brain dynamics across wake and sleep
Source: Sci Rep. 2024 Mar 29;14:7531. doi: 10.1038/s41598-024-58123-6 (PMC10980824; doi:10.1038/s41598-024-58123-6)
Supplement: Supplementary file 1 — Supplementary Information. [file 41598_2024_58123_MOESM1_ESM.pdf]

Motor learning- and consolidation-related resting state fast and slow brain dynamics across wake and sleep

Supplementary information

Liliia Roshchupkina [1-3]*, Vincent Wens [2-4], Nicolas Coquelet [2-4], Charline Urbain [1-3], Xavier de Tiege [2-4], Philippe Peigneux [1,2]

[1] UR2NF - Neuropsychology and Functional Neuroimaging Research Unit affiliated at CRCN - Centre for Research in Cognition and Neurosciences, Université libre de Bruxelles (ULB), Brussels, Belgium.

[2] UNI - ULB Neuroscience Institute, Brussels, Belgium.

[3] LN[2]T - Laboratoire de Neuroanatomie et Neuroimagerie translationnelles, ULB, Brussels, Belgium.

[4] Department of Functional Neuroimaging, Service of Nuclear Medicine, HUB - Hôpital Universitaire de Bruxelles, Hospital Erasme, Brussels, Belgium.

1. Best motor performance (BMP) calculation

The BMP Index was calculated by averaging for each participant the two best-performed FTT blocks in the Learning session. Table 1 presents the sequence numbers of these two FTT blocks, selected from a total of 20, representing the peak performance instances used in the BMP calculation.

Table 1. FTT blocks used for estimation of Best Motor Performance (BMP) levels

Wake group			Nap group		
Participant number	First BMP block	Second BMP block	Participant number	First BMP block	Second BMP block
1	16	19	1	17	20
2	14	15	2	19	20
3	15	18	3	17	18
4	4	5	4	17	18
5	13	14	5	11	19
6	7	9	6	13	17
7	17	18	7	4	20
8	17	18	8	9	17
9	16	18	9	13	14
10	14	16	10	16	17
11	13	19	11	15	19
12	17	19	12	12	20
13	14	15	13	5	15
14	19	20	14	4	5
			15	15	17
			16	10	20

Table 1. FTT blocks used for BMP estimation (average of the two FTT blocks with the best performance) in the Learning session.

2. Evolution of motor performance (non-normalised, raw data)

The learning curves observed during the session for both groups aligned with previous findings [1] showcasing a rapid initial improvement that eventually approaches a performance plateau towards the task's conclusion. A repeated measures ANOVA computed on raw GPI scores during the learning session with within-subject factor Blocks (1 to 20) and Group (Nap vs. Wake) factors disclosed a main Blocks effect (F(19,532) = 15.69, p < .001; Figure 1A) with gradually improving performance over practice. The Group effect was significant (F(1,28) = 13,05, p < .001), with on average superior performance levels in the Nap than in the Wake group. However, the Group X Session interaction (F(19,552) = 1.33, p = .16) effect was non-significant, indicating a similar evolution of behavioural performance despite the initial between-group differences.

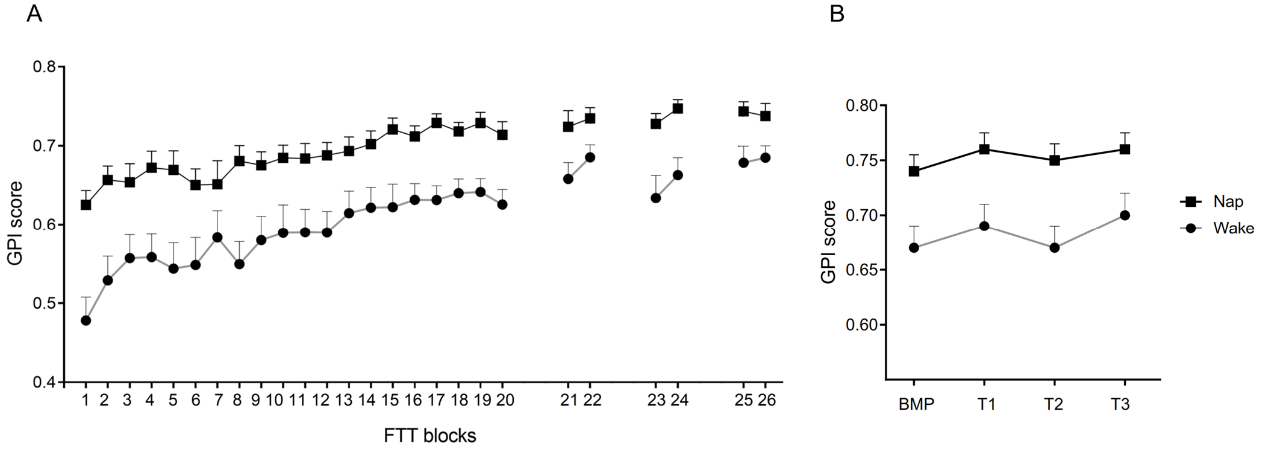

Figure 1. Global Performance Index (GPI) evolution. **A.** Mean GPI scores across 26 blocks of Finger Tapping Task (FTT); blocks 1-20 correspond to the Learning session; blocks 21-22 – boost (T1); blocks 23-24 – silent (T2) and blocks 25-26 – the next day (T3) windows. **B.** GPI evolution from Best Motor Performance (BMP) achieved in the Learning session to each testing session. Circles: Wake condition; Squares: Nap condition. Error bars represent standard errors.

The evolution of offline performance assessed using repeated measures ANOVA on raw GPI scores with Session (BMP x Test 1 x Test 2 x Test 3) and Group (Nap vs. Wake) factors revealed a significant main effect for Session (F(3,84) = 5.45, p = .002), indicating notable changes over time. Subsequent post-hoc analyses elucidated a temporal progression in performance gains, notably during the boost (BMP vs. T1: t(29) = -3.72, p = .001) and the next day (BMP vs. T3: t(29) = -3.34, p = .002) periods. Additionally, a noticeable improvement was observed from the silent phase to the following day (T2 vs. T3: t(29) = -2.06, p = .05). However, the interaction between Group and Session was not significant (F(3,84) = 0.78, p = .51; Figure 1B), suggesting the observed temporal patterns were consistent across groups. Again, a significant Group main effect was evidenced (F(1,

28) = 12.74, *p* = .001). Due to these unexpected between-group differences in the pre-experimental manipulation Learning session, normalized (Z-zcore) GPI data are reported in the main manuscript.

3. Sleep measures

Polysomnographic data (naps and nights) were scored using 20-sec epochs with bandpass 0.3-30 Hz for the scalp electrodes.

Table 2. Sleep scoring in the 90-min nap conditions

Sleep Stage	Nap sleep (min)	Nap sleep (%)
Wake	12.31 ± 3.30 (46.67)	13.59 ± 3.59 (49.08)
NREM 1	9.88 ± 1.00 (14.66)	11.01 ± 1.12 (16.30)
NREM 2	28.17 ± 3.04 (47.33)	31.37 ± 3.37 (52.99)
SWS	32.98 ± 4.40 (60.33)	36.85 ± 4.96 (66.77)
REM	6.87 ± 2.33 (16.33)	12.75 ± 2.68 (26.56)
TST	77.46 ± 3.11 (43.00)	86.41 ± 3.59 (43.15)

Table 2. Sleep scoring in the 90-min nap conditions. Mean values and standard errors indicate the duration (min) and percentage (%) of time spent in each stage; the range is indicated in brackets. The range of data is in brackets. Abbreviations: NREM (non-rapid eye movement sleep), SWS (slow wave sleep), REM (rapid-eye movement sleep), TST (total sleep time, considers all sleep stages except wakefulness).

Table 3. Nocturnal sleep scorings in the Wake and Nap groups

Sleep Stage	Wake group (min)	Wake group (%)	Nap group (min)	Nap group (%)
Wake	21.83 ± 5.81 (76.66)	4.75 ± 1.27 (17.01)	33.53 ± 10.19 (121.33)	7.26 ± 2.11 (25.29)
NREM 1	28.67 ± 3.20 (43.00)	6.21 ± 0.66 (8.37)	36.71 ± 4.38 (52.66)	8.34 ± 1.07 (14.57)
NREM 2	133.86 ± 12.73 (159.00)	28.88 ± 2.52 (29.83)	136.05 ± 9.49 (138.00)	30.83 ± 2.25 (31.05)
SWS	178.10 ± 12.06 (144.67)	38.96 ± 2.75 (31.00)	150.43 ± 8.83 (126.33)	33.75 ± 1.72 (22.36)
REM	97.96 ± 6.22 (88.00)	21.19 ± 1.19 (17.94)	88.26 ± 4.88 (48.33)	19.81 ± 0.97 (9.89)
TST	438.58 ± 10.25 (91.00)	95.25 ± 1.27 (17.23)	411.45 ± 10.58 (125.67)	92.74 ± 2.11 (25.29)

Table 3. Nocturnal sleep scorings in the Wake and Nap groups. The mean values and standard errors indicate the duration (min) and percentage (%) of time spent in each stage; the range is indicated in brackets. Abbreviations: NREM (non-rapid eye movement sleep), SWS (slow wave sleep), REM (rapid-eye movement sleep), TST (total sleep time, considers all sleep stages except wakefulness).

4. Sleep macrostructure and performance

To examine the connections between sleep macrostructure and performance, we performed correlational analyses linking the duration of nap and nighttime sleep (as detailed in Tables 4 and 5) with normalised BMP/LI data.

Table 4. Correlations between duration of nap sleep stages and behavioural indices (normalised data).

Sleep Stage Nap	BMP		LI	
	Pearson's r	*p* - value	Pearson's r	*p* - value
Wake	0.06	0.84	-0.07	0.79
NREM 1	-0.05	0.86	0.05	0.84
NREM 2	0.35	0.19	0.16	0.56
SWS	-0.37	0.16	-0.21	0.43
REM	0.20	0.48	0.23	0.41
TST	-0.08	0.77	0.03	0.90

Table 4. Correlations between duration of nap sleep stages and behavioural (normalized, Z-score) indices. Abbreviations: NREM (non-rapid eye movement sleep), SWS (slow wave sleep), REM (rapid-eye movement sleep), TST (total sleep time, considers all sleep stages except wakefulness), BMP (Best motor performance), LI (Learning index).

Table 5. Correlations between duration of night sleep stages and behavioural indices (normalised data).

Sleep Stage	Wake				Nap			
	BMP		LI		BMP		LI	
	Pearson's r	*p* - value	Pearson's r	*p* - value	Pearson's r	*p* - value	Pearson's r	*p* - value
Wake	0.23	0.44	0.12	0.69	-0.16	0.58	-0.07	0.81
NREM 1	0.23	0.42	0.17	0.56	-0.29	0.32	-0.05	0.88
NREM 2	-0.22	0.46	-0.34	0.24	0.26	0.37	-0.33	0.25
SWS	0.03	0.93	0.24	0.41	0.08	0.78	0.33	0.26
REM	-0.05	0.88	-0.29	0.31	-0.11	0.71	0.23	0.42
TST	-0.19	0.51	-0.26	0.37	0.13	0.65	0.06	0.83

Table 5. Correlations between duration of night sleep stages and behavioural (normalized, Z-score) indices. Abbreviations: NREM (non-rapid eye movement sleep), SWS (slow wave sleep), REM (rapid-eye movement sleep), TST (total sleep time, considers all sleep stages except wakefulness), BMP (Best motor performance), LI (Learning index).

5. HMM temporal characteristics

To reveal the impact of motor learning (ML) on HMM temporal parameters, we performed a repeated measures ANOVA with Sessions (T1 vs. T2 vs. T3) and Induction (pre – vs. post-behavioural testing) as within-subject factors and Group (Nap vs. Wake) as between-subjects factor. The significance level was set at $p < .05$, Bonferroni corrected by a factor of 21 (i.e., 7 independent HMM states and 3 independent HMM temporal parameters).

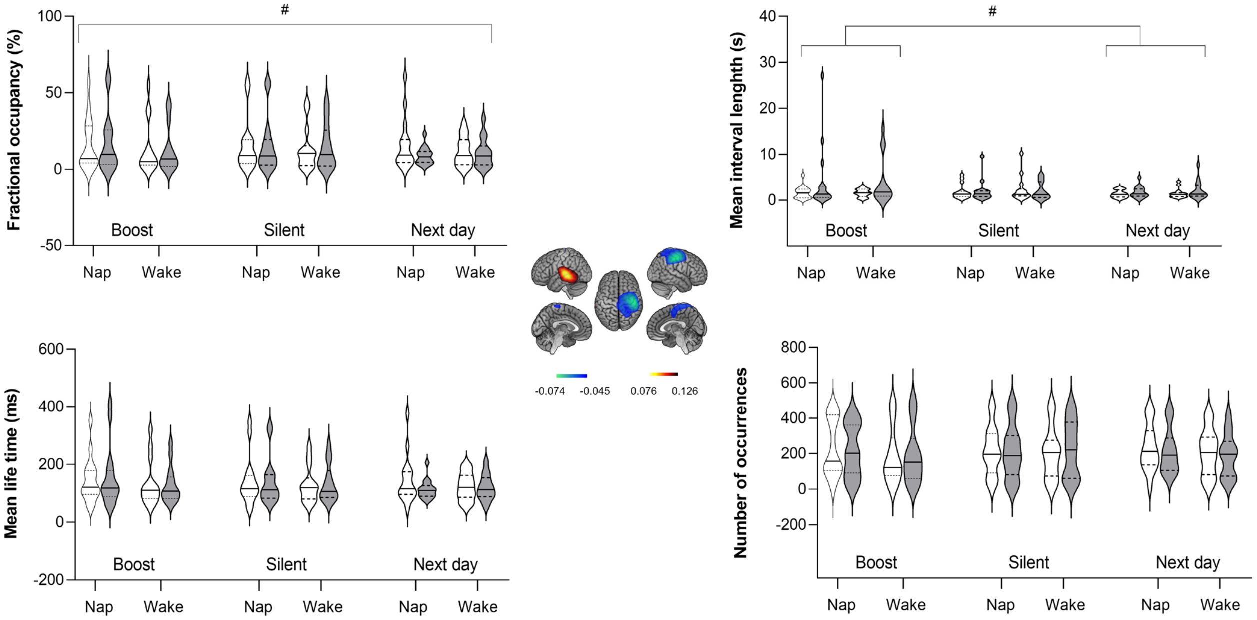

Figure 2. HMM temporal parameters for State 3 (Temporal/Sensorimotor). White violins represent non-induced RS sessions, grey violins – represent induced RS, and the medians are in solid lines and quartiles - dotted lines. ($^{\#}p \leq .05$ not corrected for multiple comparisons).

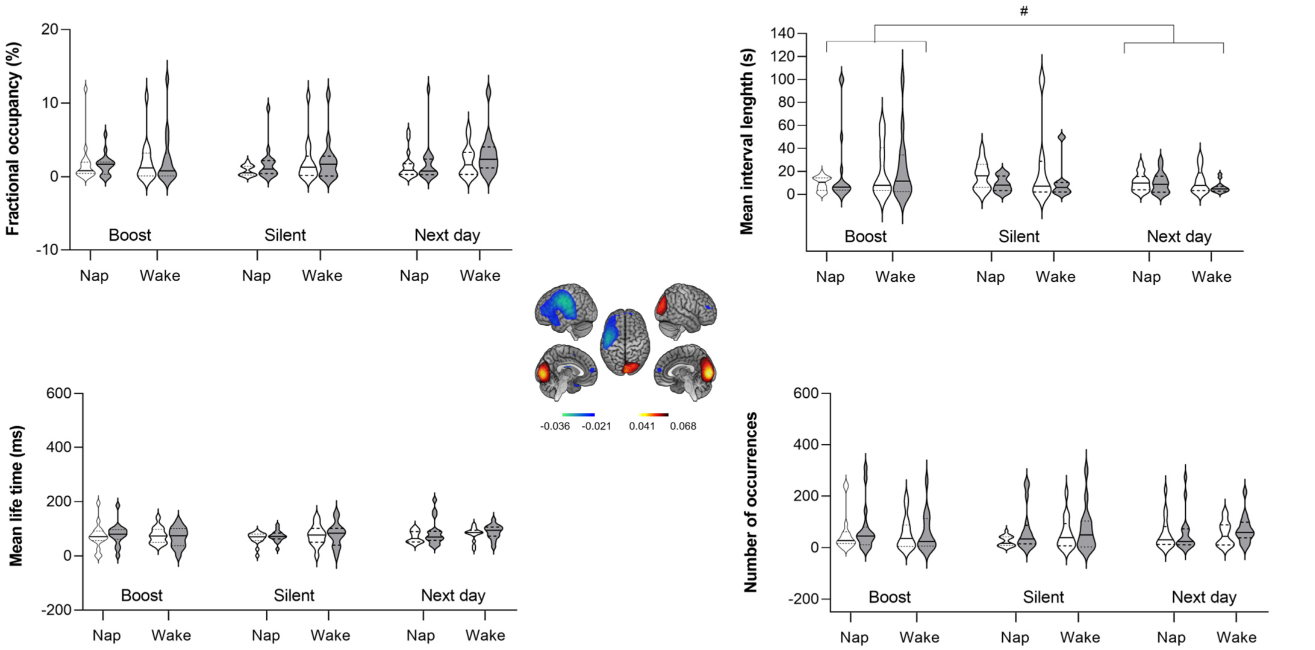

Figure 3. HMM temporal parameters for State 5 (Calcarine/Postcentral). White violins represent non-induced RS sessions, grey violins – represent induced RS, and the medians are in solid lines and quartiles - dotted lines; ($^{\#}p \leq .05$ not corrected for multiple comparisons).

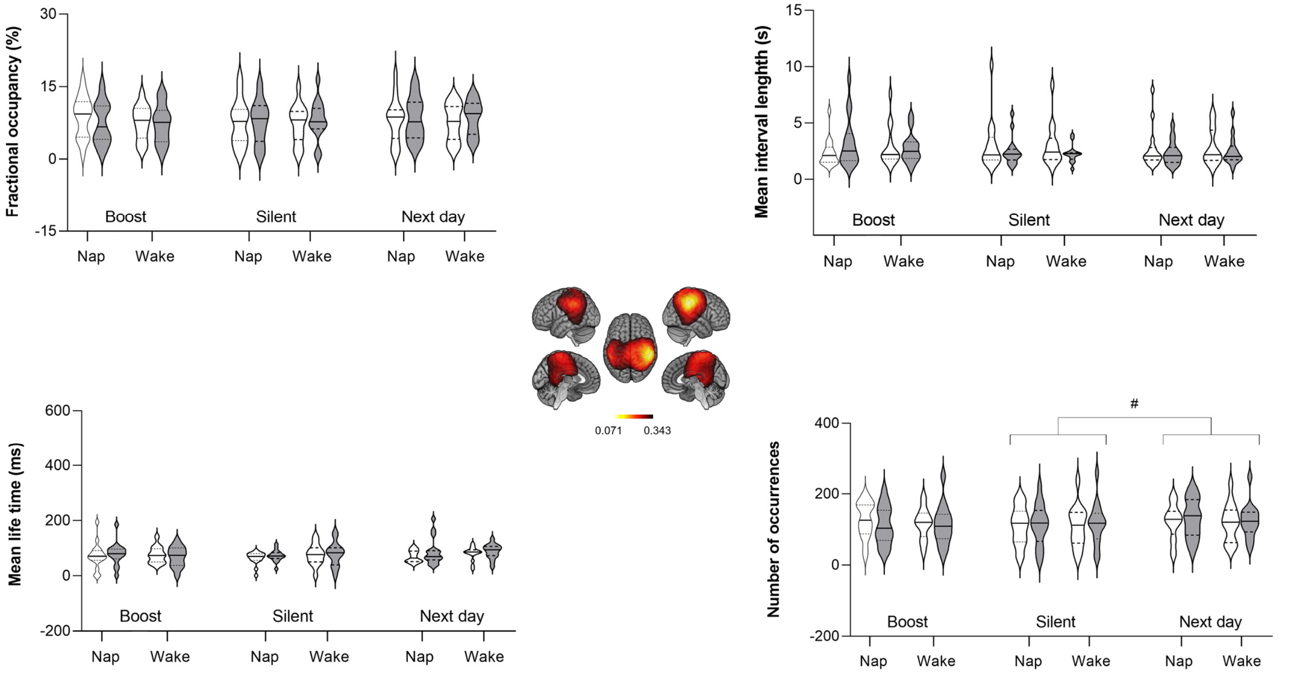

Figure 4. HMM temporal parameters for State 6 (Supramarginal). White violins represent non-induced RS sessions, grey violins – represent induced RS, and the medians are in solid lines and quartiles - dotted lines. ($^{\#}p \leq .05$ not corrected for multiple comparisons).

Table 6 represents the results of repeated measures ANOVA for States 3, 4, 5 and 6. Figures 2-4 illustrate the results for these HMM states.

Table 6. HMM temporal parameters (repeated measures ANOVA) results

**Sate 3**				
	FO	MLT	NO	MIL
Session	F = 3.16<br>**$p = 0.05^{\#}$**	F = 2.91<br>$p = 0.06$	F = 0.26<br>$p = 0.77$	F = 3.54<br>**$p = 0.04^{\#}$**
Session x Group	F = 0.58<br>$p = 0.56$	F = 0.46<br>$p = 0.64$	F = 1.15<br>$p = 0.32$	F = 0.10<br>$p = 0.91$
Induction	F = 2.92<br>$p = 0.1$	F = 1.73<br>$p = 0.20$	F = 0.58<br>$p = 0.45$	F = 4.97<br>**$p = 0.03^{\#}$**
Session x Induction	F = 3.0<br>$p = 0.06$	F = 1.79<br>$p = 0.18$	F = 1.26<br>$p = 0.30$	F = 3.90<br>**$p = 0.03^{\#}$**
Group	F = 0.58<br>$p = 0.56$	F = 0.23<br>$p = 0.64$	F = 0.18<br>$p = 0.68$	F = 0.09<br>$p = 0.77$
**State 4**				
Session	F = 7.63<br>**$p = 0.001^{**}$**	F = 7.60<br>**$p = 0.001^{**}$**	F = 8.25<br>**$p < 0.001^{***}$**	F = 2.80<br>$p = 0.07$
Session x Group	F = 1.29<br>$p = 0.28$	F = 0.60<br>$p = 0.56$	F = 0.99<br>$p = 0.38$	F = 0.03<br>$p = 0.97$
Induction	F = 5.08<br>**$p = 0.03^{\#}$**	F = 2.16<br>$p = 0.15$	F = 4.52<br>**$p = 0.04^{\#}$**	F = 4.76<br>**$p = 0.04^{\#}$**
Session x Induction	F = 5.46<br>$p =$ **$0.007^{\#}$**	F = 4.22<br>**$p = 0.02^{\#}$**	F = 4.47<br>**$p = 0.02^{\#}$**	F = 3.61<br>**$p = 0.03^{\#}$**
Group	F = 0.42<br>$p = 0.52$	F = 0.20<br>$p = 0.66$	F = 0.55<br>$p = 0.46$	F = 0.08<br>$p = 0.77$
**State 5**				
Session	F = 0.47<br>$p = 0.63$	F = 1.11<br>$p = 0.34$	F = 0.18<br>$p = 0.84$	F = 4.48<br>**$p = 0.02^{\#}$**
Session x Group	F = 0.13<br>$p = 0.88$	F = 0.63<br>$p = 0.54$	F = 0.86<br>$p = 0.43$	F = 1.08<br>$p = 0.35$
Induction	F = 8.13<br>**$p = 0.008^{\#}$**	F = 5.60<br>**$p = 0.03^{\#}$**	F = 4.45<br>**$p = 0.04^{\#}$**	F = 0.54<br>$p = 0.47$
Session x Induction	F = 0.60<br>$p = 0.55$	F = 1.63<br>$p = 0.20$	F = 2.19<br>$p = 0.12$	F = 4.89<br>**$p = 0.01^{\#}$**
Group	F = 1.08<br>$p = 0.31$	F = 0.15<br>$p = 0.70$	F = 0.06<br>$p = 0.81$	F = 0.47<br>$p = 0.50$
**State 6**				
Session	F = 1.34<br>$p = 0.27$	F = 0.05<br>$p = 0.96$	F = 3.83<br>**$p = 0.03^{\#}$**	F = 0.62<br>$p = 0.54$
Session x Group	F = 0.70<br>$p = 0.50$	F = 1.65<br>$p = 0.20$	F = 0.27<br>$p = 0.76$	F = 0.21<br>$p = 0.81$
Induction	F = 0.70<br>$p = 0.50$	F = 0.35<br>$p = 0.56$	F = 0.03<br>$p = 0.86$	F = 1.47<br>$p = 0.24$

Session x Induction	F = 3.82<br>***p* = 0.03#**	F = 2.26<br>*p* = 0.12	F = 5.20<br>***p* = 0.009#**	F = 3.94<br>***p* = 0.03#**
Group	F = 0.03<br>*p* = 0.86	F = 0.01<br>*p* = 0.91	F = 0.03<br>*p* = 0.87	F = 0.01<br>*p* = 0.94

Table 6. HMM temporal parameters (repeated measure ANOVA) results for States 3, 4, 5 and 6; *p* – values (****p* < 0.001; ***p* ≤ 0.002, corrected by factor 21 (7 HMM states x 3 HMM temporal parameters); #*p* ≤ 0.05, uncorrected).

Table 7 represents the results of post-hoc analyses conducted on Induction effects within each session using a paired-sampled Wilcoxon signed-rank test for all HMM states.

Table 7. Induction effect for 8 HMM states

**Sessions**	**Fractional occupancy**		**Mean life time**		**Number of occurrences**		**Mean interval length**	
	W	*p*-values	W	*p*-values	W	*p*-values	W	*p*-values
**State 1**								
Boost	349	**0.02#**	349	**0.02#**	219	0.98	159	0.14
Silent	184	0.33	187	0.36	138.5	**0.05#**	224	0.87
Next day	196.5	0.47	245	0.81	104	**0.01#**	370	**0.004#**
**State 2**								
Boost	200	0.52	242	0.86	161	0.14	358	**0.01#**
Silent	247	0.78	228	0.94	263	0.33	286	0.28
Next day	228	0.93	207	0.61	243	0.59	322	0.07
**State 3**								
Boost	227	0.92	237	0.94	198.5	0.69	281	0.33
Silent	263	0.54	264	0.53	230	0.55	167	0.42
Next day	118	**0.03#**	173	0.23	99	**0.02#**	361	**0.01#**
**State 4**								
Boost	78	**< .001*****	115	**0.02#**	68	**< .001*****	415	**< .001*****
Silent	168	0.19	198	0.49	211	0.67	322	0.07

Next day	219	0.79	273	0.42	175.5	0.37	307	0.13
**State 5**								
Boost	193	0.67	248	0.76	223	0.42	252	0.70
Silent	303	**0.01#**	290.5	0.12	283.5	**0.02***	75	**0.001****
Next day	313	0.10	338	**0.03#**	309.5	0.12	123	**0.03#**
**State 6**								
Boost	152	0.16	214	0.72	107.5	**0.01#**	353	**0.01#**
Silent	280	0.34	276	0.38	243	0.84	159	0.13
Next day	325	0.06	328	**0.05#**	266	0.30	158	0.13
**State 7**								
Boost	329	**0.05#**	263	0.54	245.5	0.55	200	0.52
Silent	98	**0.01#**	158	0.20	111.5	**0.02#**	235	0.97
Next day	110	**0.02#**	232	1.00	107	**0.01#**	352	**0.01#**
**State 8**								
Boost	354	**0.01#**	344	**0.02#**	289.5	0.12	93	**0.004#**
Silent	265	0.52	261	0.57	219.5	0.97	217	0.76
Next day	265	0.52	318	0.08	191	0.57	200	0.96

Table 7. Induction effect for 8 HMM states. W - Wilcoxon signed-rank; (****p* < 0.001; ***p* ≤ 0.002, corrected by factor 21 (7 HMM states x 3 HMM temporal parameters); #*p* ≤ 0.05, uncorrected).

6. Resting-state functional connectivity (rsFC) wideband

The functional connectome of our study included 126 regions of interest (116 nodes correspond to the Automated Anatomical Labelling (AAL) atlas [2] (including the cerebellum). Additionally, we added ten nodes based on the relevant learning/memory literature (see Table 8). The MNI coordinates from the literature were derived/adjusted using the SPM Anatomy toolbox, Version 2.2b. To identify the network of regions engaged in motor learning and consolidation, we computed the adjacency matrix (126 by 126) for wideband (4–30 Hz). A statistical within-group comparison of inter-regional connectivity differences was then computed on the adjacency matrix using nonparametric Network Based Statistics (NBS) [3], [4]. Our results reveal that re-introducing the task (induction effect) resulted in a vast neural network in the boost window (RS 3 vs. RS 2) comprising 39 edges and 28 nodes *p* = 0.038. Table 9 represents the nodes list of this emerged network.

Table 8. Full connectome list of nodes

Label	x	y	z	Region	Reference
1	-39	-6	51	Precentral_L	AAL
2	41	-8	52	Precentral_R	AAL
3	-18	35	42	Frontal_Sup_L	AAL
4	22	31	44	Frontal_Sup_R	AAL
5	-17	47	-13	Frontal_Sup_Orb_L	AAL
6	18	48	-14	Frontal_Sup_Orb_R	AAL
7	-33	33	35	Frontal_Mid_L	AAL
8	38	33	34	Frontal_Mid_R	AAL
9	-31	50	-10	Frontal_Mid_Orb_L	AAL
10	33	53	-11	Frontal_Mid_Orb_R	AAL
11	-48	13	19	Frontal_Inf_Oper_L	AAL
12	50	15	21	Frontal_Inf_Oper_R	AAL
13	-46	30	14	Frontal_Inf_Tri_L	AAL
14	50	30	14	Frontal_Inf_Tri_R	AAL
15	-36	31	-12	Frontal_Inf_Orb_L	AAL
16	41	32	-12	Frontal_Inf_Orb_R	AAL
17	-47	-8	14	Rolandic_Oper_L	AAL
18	53	-6	15	Rolandic_Oper_R	AAL
19	-5	5	61	Supp_Motor_Area_L	AAL
20	9	0	62	Supp_Motor_Area_R	AAL
21	-8	15	-11	Olfactory_L	AAL
22	10	16	-11	Olfactory_R	AAL
23	-5	49	31	Frontal_Sup_Medial_L	AAL
24	9	51	30	Frontal_Sup_Medial_R	AAL
25	-5	54	-7	Frontal_Med_Orb_L	AAL
26	8	52	-7	Frontal_Med_Orb_R	AAL
27	-5	37	-18	Rectus_L	AAL
28	8	36	-18	Rectus_R	AAL
29	-35	7	3	Insula_L	AAL
30	39	6	2	Insula_R	AAL
31	-4	35	14	Cingulum_Ant_L	AAL
32	8	37	16	Cingulum_Ant_R	AAL
33	-5	-15	42	Cingulum_Mid_L	AAL
34	8	-9	40	Cingulum_Mid_R	AAL
35	-5	-43	25	Cingulum_Post_L	AAL
36	7	-42	22	Cingulum_Post_R	AAL
37	29	-20	-10	Hippocampus_R	AAL
38	-25	-21	-10	Hippocampus_L	AAL
39	-21	-19	-23	ParaHippocampal_L	AAL
40	21	-19	-23	ParaHippocampal_R	AAL
41	-23	-1	-17	Amygdala_L	AAL
42	27	1	-18	Amygdala_R	AAL
43	-7	-79	6	Calcarine_L	AAL
44	16	-73	9	Calcarine_R	AAL
45	-6	-80	27	Cuneus_L	AAL

46	14	-79	28	Cuneus_R	AAL
47	-15	-68	-5	Lingual_L	AAL
48	16	-67	-4	Lingual_R	AAL
49	-17	-84	28	Occipital_Sup_L	AAL
50	24	-81	31	Occipital_Sup_R	AAL
51	-32	-81	16	Occipital_Mid_L	AAL
52	37	-80	19	Occipital_Mid_R	AAL
53	-36	-78	-8	Occipital_Inf_L	AAL
54	38	-82	-8	Occipital_Inf_R	AAL
55	-31	-40	-20	Fusiform_L	AAL
56	34	-39	-20	Fusiform_R	AAL
57	-42	-23	49	Postcentral_L	AAL
58	41	-25	53	Postcentral_R	AAL
59	-23	-60	59	Parietal_Sup_L	AAL
60	26	-59	62	Parietal_Sup_R	AAL
61	-43	-46	47	Parietal_Inf_L	AAL
62	46	-46	50	Parietal_Inf_R	AAL
63	-56	-34	30	SupraMarginal_L	AAL
64	58	-32	34	SupraMarginal_R	AAL
65	-44	-61	36	Angular_L	AAL
66	46	-60	39	Angular_R	AAL
67	-7	-56	48	Precuneus_L	AAL
68	10	-56	44	Precuneus_R	AAL
69	-8	-25	70	Paracentral_Lobule_L	AAL
70	7	-32	68	Paracentral_Lobule_R	AAL
71	-11	11	9	Caudate_L	AAL
72	15	12	9	Caudate_R	AAL
73	-24	4	2	Putamen_L	AAL
74	28	5	2	Putamen_R	AAL
75	-18	0	0	Pallidum_L	AAL
76	21	0	0	Pallidum_R	AAL
77	-11	-18	8	Thalamus_L	AAL
78	13	-18	8	Thalamus_R	AAL
79	-42	-19	10	Heschl_L	AAL
80	46	-17	10	Heschl_R	AAL
81	-53	-21	7	Temporal_Sup_L	AAL
82	58	-22	7	Temporal_Sup_R	AAL
83	-40	15	-20	Temporal_Pole_Sup_L	AAL
84	48	15	-17	Temporal_Pole_Sup_R	AAL
85	-56	-34	-2	Temporal_Mid_L	AAL
86	57	-37	-1	Temporal_Mid_R	AAL
87	-36	15	-34	Temporal_Pole_Mid_L	AAL
88	44	15	-32	Temporal_Pole_Mid_R	AAL
89	-50	-28	-23	Temporal_Inf_L	AAL
90	54	-31	-22	Temporal_Inf_R	AAL
91	-35	-67	-29	Cerebellum_Crus1_L	AAL
92	38	-67	-30	Cerebellum_Crus1_R	AAL
93	-28	-73	-38	Cerebellum_Crus2_L	AAL

94	33	-69	-40	Cerebellum_Crus2_R	AAL
95	-8	-37	-19	Cerebellum_3_L	AAL
96	13	-34	-19	Cerebellum_3_R	AAL
97	-14	-43	-17	Cerebellum_4_5_L	AAL
98	18	-43	-18	Cerebellum_4_5_R	AAL
99	-22	-59	-22	Cerebellum_6_L	AAL
100	26	-58	-24	Cerebellum_6_R	AAL
101	-31	-60	-45	Cerebellum_7b_L	AAL
102	34	-63	-48	Cerebellum_7b_R	AAL
103	-25	-55	-48	Cerebellum_8_L	AAL
104	26	-56	-49	Cerebellum_8_R	AAL
105	-10	-49	-46	Cerebellum_9_L	AAL
106	10	-49	-46	Cerebellum_9_R	AAL
107	-22	-34	-42	Cerebellum_10_L	AAL
108	27	-34	-41	Cerebellum_10_R	AAL
109	2	-39	-20	Vermis_1_2	AAL
110	2	-40	-11	Vermis_3	AAL
111	2	-52	-6	Vermis_4_5	AAL
112	2	-67	-15	Vermis_6	AAL
113	2	-72	-25	Vermis_7	AAL
114	2	-64	-34	Vermis_8	AAL
115	2	-55	-35	Vermis_9	AAL
116	1	-46	-32	Vermis_10	AAL
117	-34	-76	49	Parietal_Inf_Lobe_L	[5]
118	-4	28	42	Superior_Medial_Gyrus_L	[6]
119	28	-10	-37	Enthorinal_cortex_R	[7]
120	-23	-8	-38	Enthorinal_cortex_L	[7]
121	-1	-17	55	Posterior_Medial_Frontal_L (SMA_L)	[8]
122	8	-17	57	Posterior_Medial_Frontal_R (SMA_R)	[8]
123	-17	-58	-38	Dentate_Nucleus_L	[9]
124	14	-61	-38	Dentate_Nucleus_R	[9]
125	11	-12	0	Subthalamic_Nucleus_R	[10]
126	-8	-13	-7	Subthalamic_Nucleus_L	[10]

Table 8. Full connectome node list (116 AAL nodes (not highlighted); 10 additional nodes inspired by literature (in grey). The MNI coordinates of additional nodes derived/were checked with the Anatomy toolbox (SPM).

Table 9. The node list of the wideband rsFC network for the induction effect (Rest 3 vs. Rest 2) in the boost time window

Number	Node's weight	Region
1	9.0	Precentral_L
2	7.2	Frontal_Inf_Oper_R
3	3.9	Rolandic_Oper_R
4	19.4	Supp_Motor_Area_R
5	25.9	Insula_L
6	7.3	Cingulum_Mid_R
7	3.9	Cingulum_Post_R
8	3.8	Occipital_Sup_L
9	11.6	Occipital_Mid_L
10	7.4	Occipital_Mid_R
11	8.6	Postcentral_L
12	7.8	Parietal_Inf_L
13	3.9	Angular_R
14	7.4	Precuneus_L
15	3.8	Precuneus_R
16	3.6	Paracentral_Lobule_L
17	7.4	Paracentral_Lobule_R
18	3.6	Putamen_R
19	7.2	Pallidum_R
20	3.6	Heschl_L
21	31.3	Temporal_Sup_L
22	39.8	Temporal_Mid_L
23	10.8	Cerebellum_Crus2_L
24	10.8	Cerebellum_6_L
25	7.2	Cerebellum_6_R
26	7.2	Vermis_7
27	7.7	Posterior_Medial_Frontal _R (SMA_R)
28	26.8	Dentate_Nucleus_L

Table 9. The node list of the wideband resting-state functional connectivity (rsFC) analysis for the induction effect (Rest 3 vs. Rest 2) in the boost time window.

7. Resting-state functional connectivity 5-band data

To reveal band-specific rsFC changes induced by motor learning, we computed the adjacency matrix (126 by 126) for five distinct frequency bands: delta (δ 1–4 Hz), theta (θ band: 4–8 Hz), alpha (α band: 8–13 Hz), beta (β band: 13–30 Hz) and gamma (γ band: 30–45 Hz). Next, we performed a statistical within-group comparison of inter-regional connectivity differences as described for the wideband in the previous section. The results of this analysis are illustrated in Figures 5 - 11.

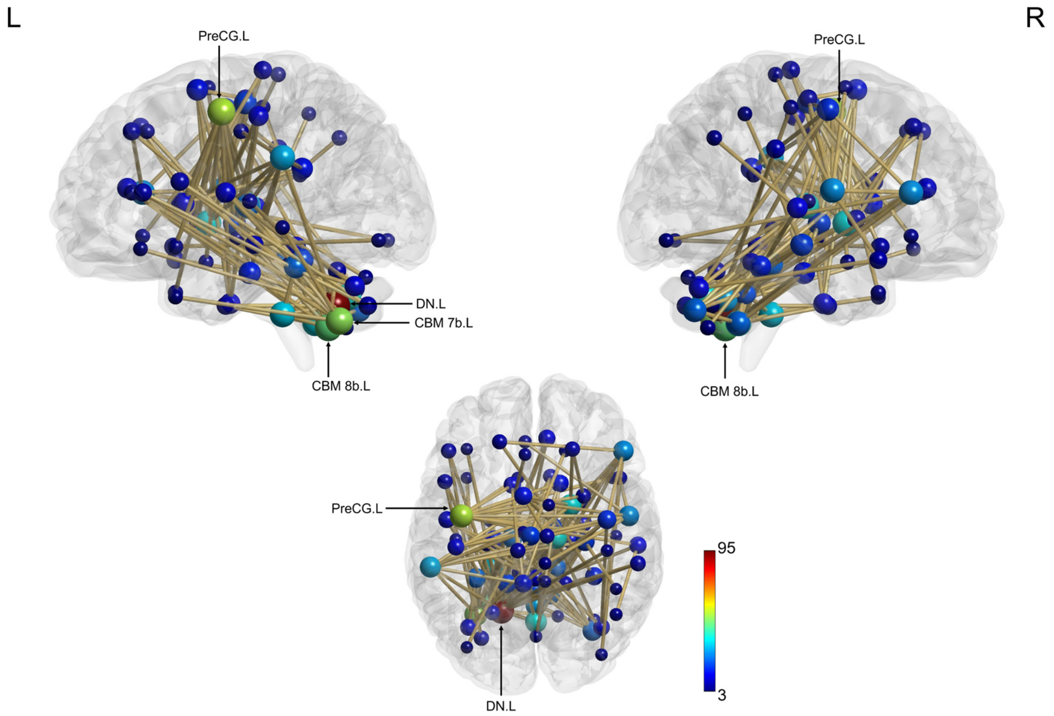

Figure 5. Neural network representing increased connectivity in post-learning session (Rest 2 vs. Rest 1) in the theta band. Nodes are scaled according to their weight (the sum of all edges connected to the node). Significant edges are represented as interconnecting lines between 126 connectome seed regions. Abbreviations: PreCG – Precentral gyrus; DN – Dentate nucleus. L – left; R – right.

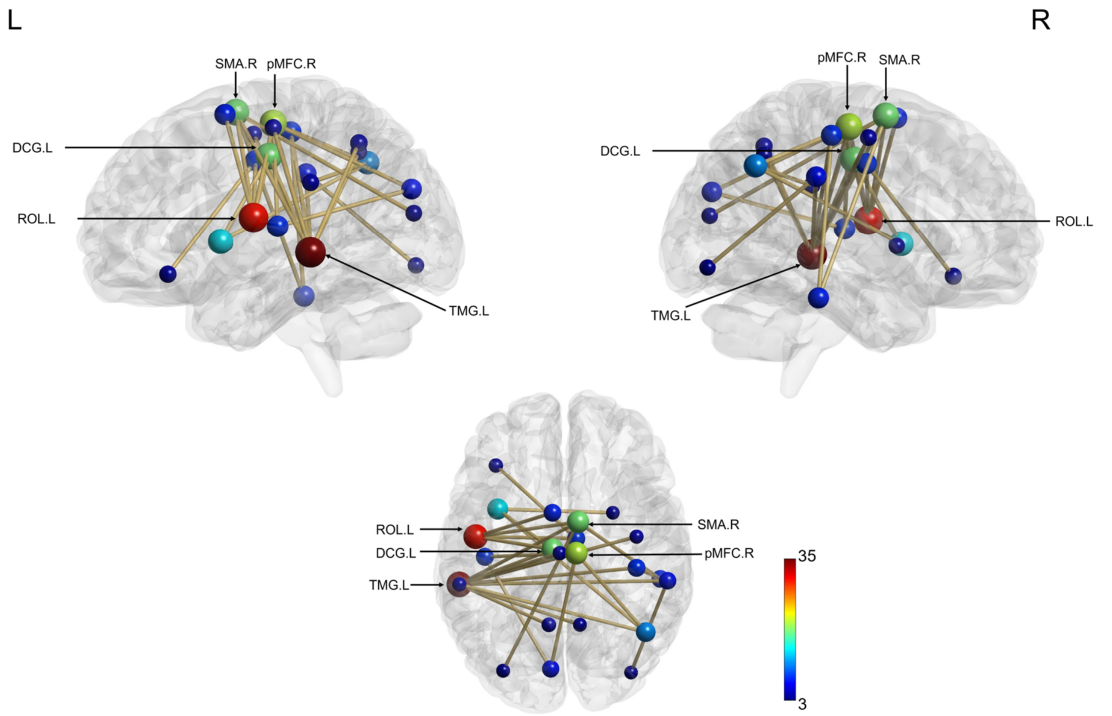

Figure 6. Neural network representing the increased connectivity in the alpha band as a result of induction effect (re-introduction to the task) during the boost period. Nodes are scaled according to their weight (the sum of all edges connected to the node). Significant edges are represented as interconnecting lines between 126 connectome seed regions. Abbreviations: ROL – Rolandic operculum; TMG – Temporal middle gyrus; pMFC – posterior Medial frontal cortex; SMA – Supplementary motor area; DCG – Median cingulate and paracingulate gyri; L – left; R – right.

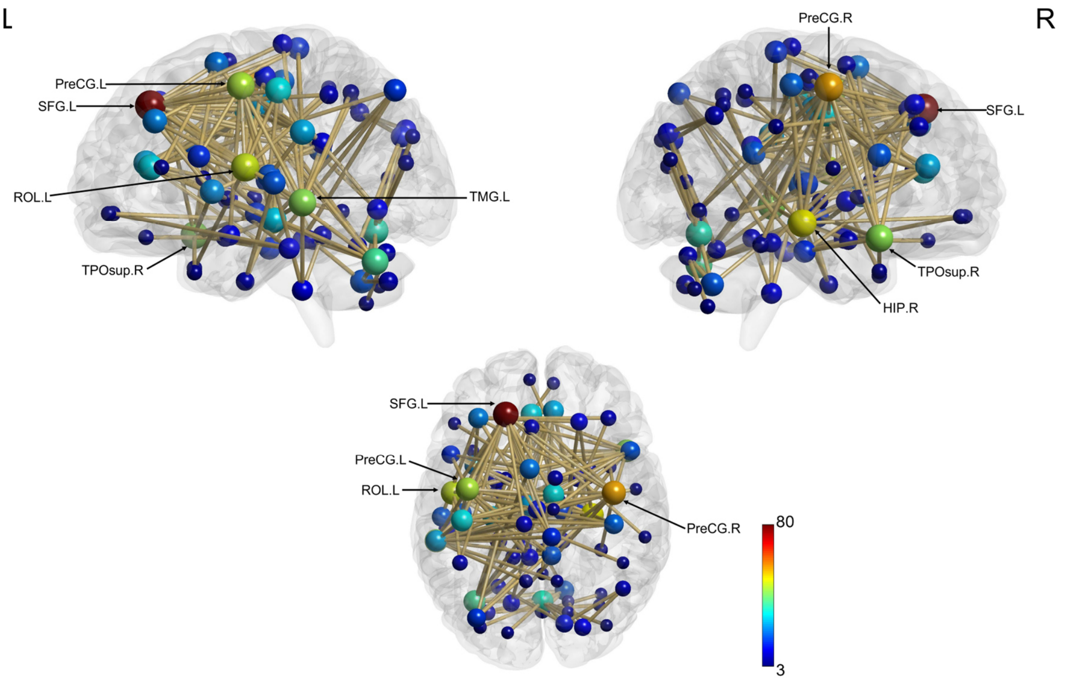

Figure 7. Neural network representing the increased connectivity in the beta band as a result of induction effect (re-introduction to the task) during the boost period. Nodes are scaled according to their weight (the sum of all edges connected to the node). Significant edges are represented as interconnecting lines between 126 connectome seed regions. Abbreviations: SFG – Superior frontal gurus; PreCG – Precentral gyrus; ROL – Rolandic operculum; L – left; R – right.

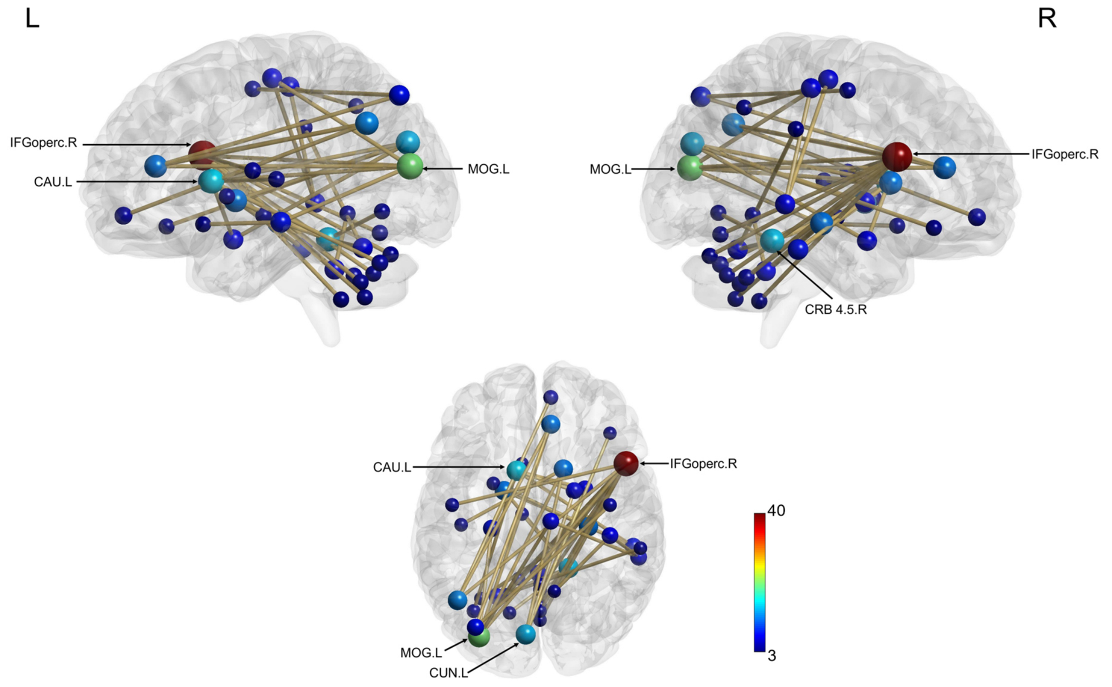

Figure 8. Neural network representing the increased connectivity in the delta band as a result of induction effect (re-introduction to the task) during the boost period. Nodes are scaled according to their weight (the sum of all edges connected to the node). Significant edges are represented as interconnecting lines between 126 connectome seed regions. Abbreviations: IFGoperc – Inferior frontal gyrus, opercular part; MOG – Middle occipital gyrus; CAU – Caudate nucleus; CUN – Cuneus; L – left; R – right.

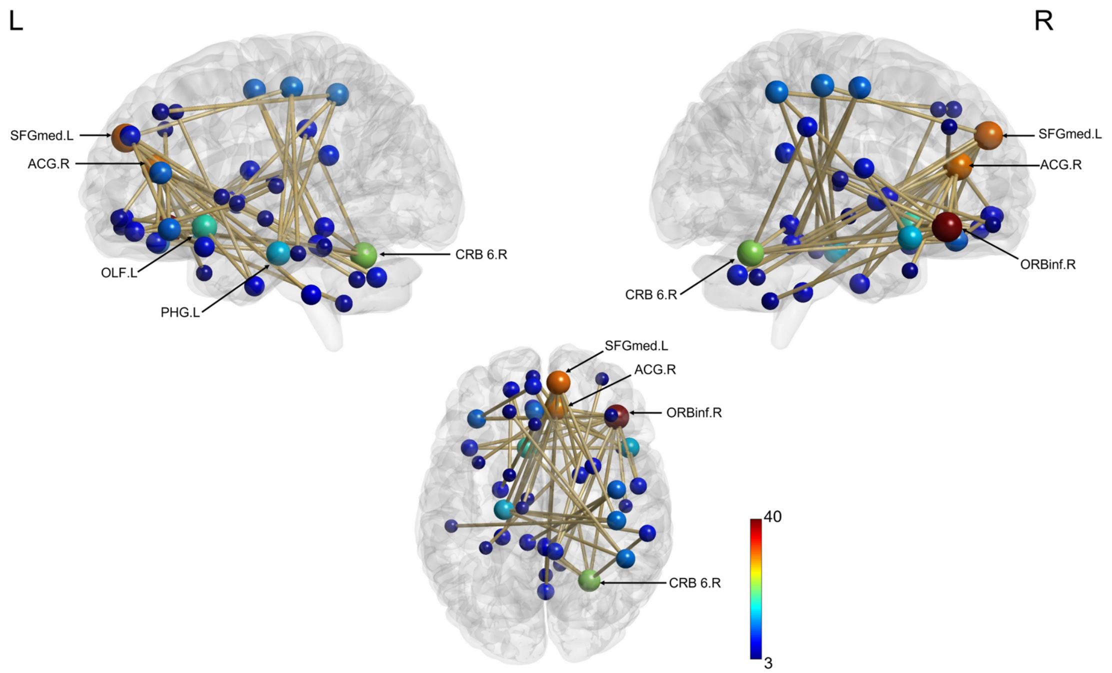

Figure 9. Neural network representing the increased connectivity in gamma band as a result of induction effect (re-introduction to the task) during the boost period. Nodes are scaled according to their weight (the sum of all edges connected to the node). Significant edges are represented as interconnecting lines between 126 connectome seed regions. Abbreviations: ORBinf – Inferior frontal gyrus, orbital part; ACG – Anterior cingulate and paracingulate gyri; SFGmed – Superior frontal gyrus, medial; CRB 6 – Cerebellum lobe 6; L – left; R – right.

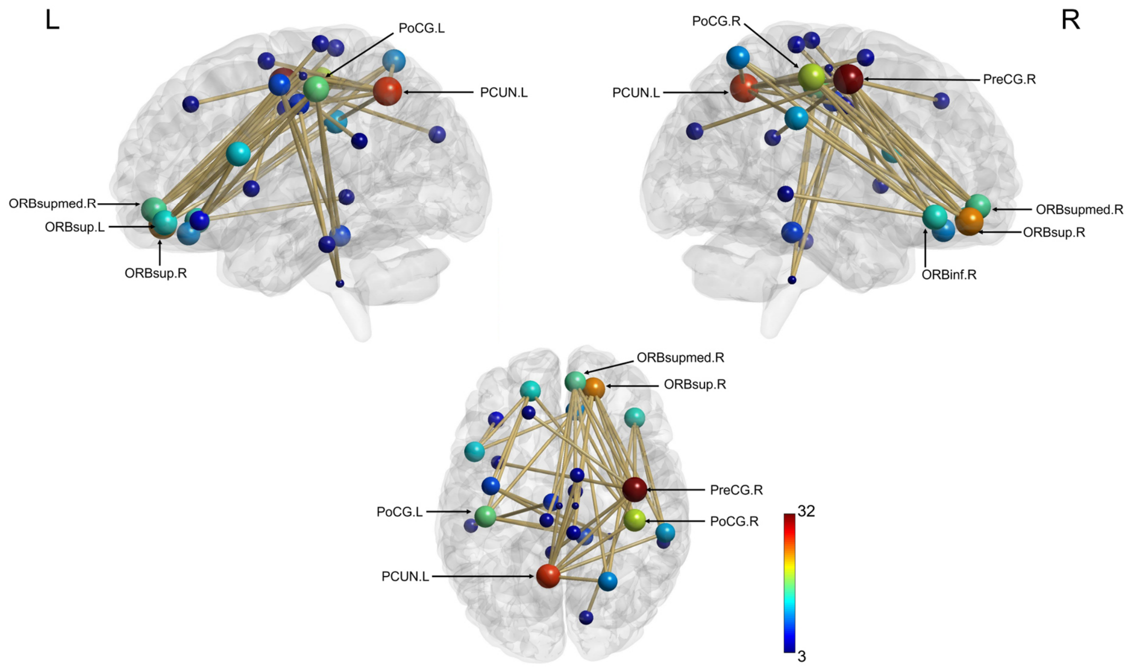

Figure 10. Neural network representing the increased connectivity in alpha band as a result of induction effect (re-introduction to the task) during the next day period. Nodes are scaled according to their weight (the sum of all edges connected to the node). Significant edges are represented as interconnecting lines between 126 connectome seed regions. Abbreviations: PreCG – Precental gyrus; PCUN – Precuneus; ORBsup – Superior frontal gyrus, orbital part; PoCG – Postcentral gyrus; ORBsupmed – Superior frontal gyrus, medial orbital; L – left; R – right.

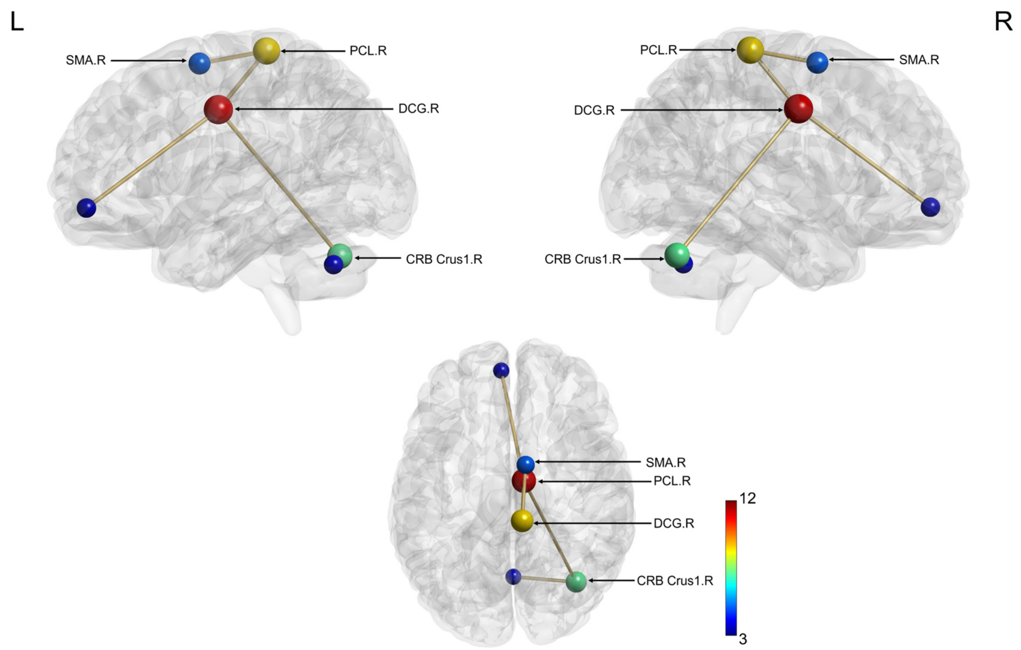

Figure 11. Neural network representing the increased connectivity in gamma band as a result of induction effect (re-introduction to the task) during the next day period. Nodes are scaled according to their weight (the sum of all edges connected to the node). Significant edges are represented as interconnecting lines between 126 connectome seed regions. Abbreviations: PCL – Paracentral lobule; DCG – Median cingulate and paracingulate gyri; CRB Crus 1 – Cerebellum Crus 1; SMA – Supplementary motor area; L – left; R – right.

8. Correlational analysis between connectivity and behavioural indices

Correlational analyses between each resting-state RS session and Best Motor Performance Index (BMP) index highlighted a positive correlation between BMP and RS 3 connectivity (boost period, induced). Table 10 shows the list of nodes for the revealed resting-state functional connectivity network.

Table 10. Nodes list of the neural network which positively correlated with BMP

Number	Node's weight	Region
1	19.8	Frontal_Sup_R
2	11.3	Frontal_Mid_Orb_L
3	28.7	Frontal_Mid_Orb_R
4	9.1	Rolandic_Oper_R
5	11.6	Frontal_Med_Orb_L
6	42.3	Frontal_Med_Orb_R
7	3.9	Cingulum_Ant_L
8	3.8	ParaHippocampal_R
9	3.6	Amygdala_R
10	3.9	Fusiform_R
11	3.6	Parietal_Sup_R
12	3.9	Parietal_Inf_R
13	11.0	Angular_L
14	12.1	Angular_R
15	3.5	Paracentral_Lobule_L
16	4.3	Caudate_L
17	7.4	Thalamus_L
18	8.1	Heschl_R
19	12.3	Temporal_Sup_R
20	7.9	Temporal_Inf_R
21	3.8	Cerebellum_3_R
22	3.9	Cerebellum_4_5_R
23	4.0	Enthorinal_cortex_R
24	3.5	Subthalamic_Nucleus_R

Table 10. Nodes list of the neural network which positively correlated with Best Motor Performance (BMP).

Correlational analysis between rsFC and the Learning index (LI) revealed significant positive correlations only for RS 5 (induced RS, silent period). Table 11 lists the nodes from the emerged rsFC network.

Table 11. Nodes list of the neural network which positively correlated with the Learning index

Number	Node's weight	Region
1	9.2	Frontal_Mid_R
2	3.8	Frontal_Inf_Oper_R
3	3.8	Frontal_Inf_Tri_R
4	3.6	Frontal_Inf_Orb_L
5	10.7	Olfactory_R
6	14.6	Insula_L
7	14.6	Insula_R
8	11.5	Hippocampus_R
9	11.0	Amygdala_R
10	3.5	Calcarine_L
11	3.8	Calcarine_R
12	3.6	Cuneus_L
13	7.1	Cuneus_R
14	7.1	Lingual_R
15	3.7	Occipital_Inf_R
16	14.7	Fusiform_R
17	14.8	Caudate_L
18	3.5	Caudate_R
19	11.2	Putamen_R
20	19.9	Thalamus_R
21	8.5	Temporal_Pole_Sup_L
22	18.9	Temporal_Pole_Sup_R
23	12.0	Temporal_Pole_Mid_L
24	14.4	Cerebellum_Crus1_L
25	7.3	Cerebellum_Crus1_R
26	7.3	Cerebellum_Crus2_R
27	3.5	Cerebellum_6_L
28	3.8	Cerebellum_8_L
29	8.2	Cerebellum_9_L
30	8.2	Cerebellum_9_R
31	3.7	Vermis_3
32	11.7	Vermis_4_5
33	3.6	Vermis_6
34	7.7	Vermis_10
35	3.8	Superior_Medial_Gyrus_L
36	3.7	Dentate_Nucleus_L

Table 11. Nodes list of the neural network which positively correlated with the Learning index (LI).

9. State networks connectivity strength

Using HMM power maps, we extracted three sets of nodes (state networks) representing HMM States 3, 7, and 8 (see main text results). To quantify the connectivity strength of these state networks, we calculated the sum of all power envelope correlation values for each node within a given network. Next, we divided this sum by the number of nodes within the network to obtain a normalised measure of connectivity strength. This procedure enabled us to generate a single value for each subject in each resting-state session, which was used in a subsequent correlational analysis with behavioural measures using JASP version 0.16.2, JASP Team (2022). The results disclosed positive correlations between BMP and State 7 (Frontal/Cuneus) network. Table 12 lists the nodes comprising the network.

Table 12. The node list of the HMM state 7 (Frontal/Cuneus) network

Number	Region
1	Frontal_Sup_L
2	Frontal_Sup_R
3	Frontal_Sup_Orb_L
4	Frontal_Sup_Orb_R
5	Frontal_Mid_L
6	Frontal_Mid_R
7	Frontal_Mid_Orb_L
8	Frontal_Mid_Orb_R
9	Frontal_Inf_Tri_L
10	Frontal_Inf_Tri_R
11	Frontal_Inf_Orb_R
12	Frontal_Sup_Medial_L
13	Frontal_Sup_Medial_R
14	Frontal_Med_Orb_L
15	Frontal_Med_Orb_R
16	Rectus_L
17	Rectus_R
18	Cingulum_Ant_R

Table 12. The node list of the HMM state 7 (Frontal/Cuneus) network.

10. Fast and slow neural dynamics relationships

To reveal the relationship between fast and slow neural dynamics, we performed a correlational analysis between HMM temporal parameters and state networks' rsFC using the NBS toolbox. Table 13 illustrates the correlational outcomes for State 8.

Table 13. Correlations between HMM temporal parameters (TP) and HMM State 8 (Cuneus/Sensorimotor) network

Rest / TP	FO	MLT	NO	MIL
Rest 1	***p* = .023#** (1 edge, 2 nodes)	*p* = 1.00 (n.s)	*p* = 1.00 (n.s)	***p* = .022#** (1 edge, 2 nodes)
Rest 2	*p* = 1.00 (n.s)	*p* = 1.00 (n.s)	*p* = 1.00 (n.s)	***p* = .03#** (1 edge, 2 nodes)
Rest 3	*p* = 1.00 (n.s)	*p* = 1.00 (n.s)	***p* = .01#** (2 edges, 3 nodes)	*p* = 1.00 (n.s)
Rest 4	*p* = 1.00 (n.s)	*p* = 1.00 (n.s)	*p* = 1.00 (n.s)	*p* = 1.00 (n.s)
Rest 5	*p* = 1.00 (n.s)	*p* = 1.00 (n.s)	*p* = 1.00 (n.s)	*p* = 1.00 (n.s)
Rest 6	*p* = 1.00 (n.s)	*p* = 1.00 (n.s)	*p* = 1.00 (n.s)	*p* = 1.00 (n.s)
Rest 7	*p* = 1.00 (n.s)	*p* = 1.00 (n.s)	*p* = 1.00 (n.s)	*p* = 1.00 (n.s)

Table 13. Correlations between HMM temporal parameters (TP) and HMM State 8 (Cuneus/Sensorimotor) network. Spearman's rho and *p*-values (#*p* ≤ .05, uncorrected).

References

[1] C. Hotermans, P. Peigneux, A. M. De Noordhout, G. Moonen, and P. Maquet, "Early boost and slow consolidation in motor skill learning," *Learn. Mem.*, vol. 13, no. 5, pp. 580–583, 2006, doi: 10.1101/lm.239406.

[2] N. Tzourio-Mazoyer *et al.*, "Automated anatomical labeling of activations in SPM using a macroscopic anatomical parcellation of the MNI MRI single-subject brain," *Neuroimage*, vol. 15, no. 1, pp. 273–289, Jan. 2002, doi: 10.1006/nimg.2001.0978.

[3] A. Zalesky, A. Fornito, and E. T. Bullmore, "Network-based statistic: identifying differences in brain networks," *Neuroimage*, vol. 53, no. 4, pp. 1197–1207, Dec. 2010, doi: 10.1016/j.neuroimage.2010.06.041.

[4] A. Zalesky, L. Cocchi, A. Fornito, M. M. Murray, and E. Bullmore, "Connectivity differences in brain networks," *Neuroimage*, vol. 60, no. 2, pp. 1055–1062, Apr. 2012, doi: 10.1016/j.neuroimage.2012.01.068.

[5] M. van Elk, "The left inferior parietal lobe represents stored hand-postures for object use and action prediction," *Front. Psychol.*, vol. 5, no. APR, pp. 1–12, 2014, doi: 10.3389/fpsyg.2014.00333.

[6] S. Zhang, J. S. Ide, and C. S. R. Li, "Resting-state functional connectivity of the medial superior frontal cortex," *Cereb. Cortex*, vol. 22, no. 1, pp. 99–111, 2012, doi: 10.1093/cercor/bhr088.

[7] H. Eichenbaum, A. P. Yonelinas, and C. Ranganath, "The medial temporal lobe and recognition memory," *Annu. Rev. Neurosci.*, vol. 30, pp. 123–152, 2007, doi: 10.1146/annurev.neuro.30.051606.094328.

[8] F. De Pasquale *et al.*, "Cortical core for dynamic integration of functional networks in the resting human brain," *Neuron*, vol. 74, no. 4, pp. 753–764, 2012, doi: 10.1016/j.neuron.2012.03.031.

[9] X. Guell *et al.*, "Functional territories of human dentate nucleus," *bioRxiv*, p. 608620, Jan. 2019, doi:

10.1101/608620.

[10] B. U. Forstmann *et al.*, “Cortico-subthalamic white matter tract strength predicts interindividual efficacy in stopping a motor response,” *Neuroimage*, vol. 60, no. 1, pp. 370–375, 2012, doi: 10.1016/j.neuroimage.2011.12.044.
